# Unravelling the Regulatory Roles of lncRNAs in Melanoma: From Mechanistic Insights to Target Selection

**DOI:** 10.3390/ijms26052126

**Published:** 2025-02-27

**Authors:** Beatrice Moras, Claudia Sissi

**Affiliations:** Department of Pharmaceutical and Pharmacological Sciences, University of Padova, Via Marzolo 5, 35131 Padova, Italy; beatricemoras98@gmail.com

**Keywords:** melanoma, lncRNA, chromatin remodeling, transcriptional regulation, translational regulation

## Abstract

Melanoma is the deadliest form of skin cancer, and its treatment poses significant challenges due to its aggressive nature and resistance to conventional therapies. Long non-coding RNAs (lncRNAs) represent a new frontier in the search for suitable targets to control melanoma progression and invasiveness. Indeed, lncRNAs exploit a wide range of regulatory functions along chromatin remodeling, gene transcription, post-transcription, transduction, and post-transduction to ultimately tune multiple cellular processes. The understanding of this intricate and flexible regulatory network orchestrated by lncRNAs in pathological conditions can strategically support the rational identification of promising targets, ultimately speeding up the setup of new therapeutics to integrate the currently available approaches. Here, the most recent findings on lncRNAs involved in melanoma will be analyzed. In particular, the functional links between their mechanisms of action and some frequently underestimated features, like their different subcellular localizations, will be highlighted.

## 1. Introduction

Melanoma is a type of malignant tumor that arises from the uncontrolled proliferation of melanocytes. These cells are located in the basal layer of the epidermis, where they are responsible for the production of melanin. Since they derive from the neuronal crest, melanocytes are not only present in the skin but are also found in various other tissues throughout the body. Consequently, melanomas can be classified into two main groups: cutaneous and non-cutaneous. Within the non-cutaneous category, we can further distinguish mucosal melanoma, which occurs in areas such as the mouth, vagina, anus, and rectum, and uveal melanoma, which develops in the uvea of the eye [1]. Distinct from the non-cutaneous melanoma, cutaneous melanoma has a significantly higher incidence [2]. Indeed, the World Health Organization reported 331,722 new cases of melanoma and 58,667 deaths in 2022, ranking it as the 17th most common cancer worldwide [3,4,5].

**Figure 1:**
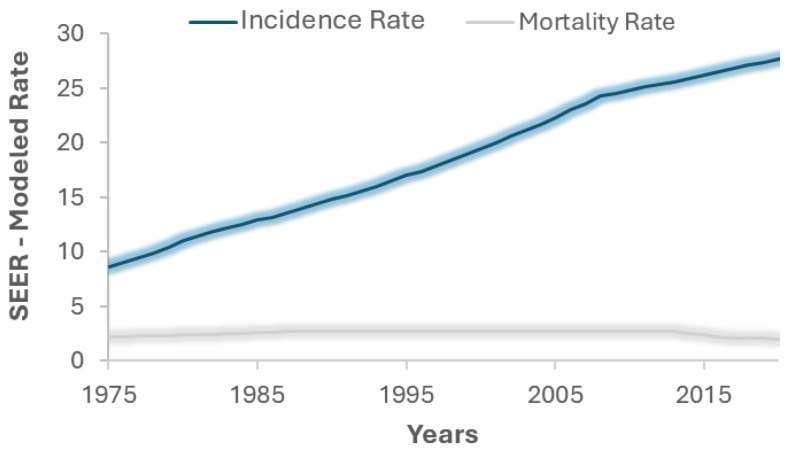
Melanoma incidence and mortality rates from 1975 to 2020 derived from the Surveillance, Epidemiology, and End Results (SEER) program.

The incidence of cancers can be effectively understood through a statistical analysis of data recorded over the years by the Surveillance, Epidemiology, and End Results (SEER) program. This program gathers information on cancer incidence and survival from approximately 34.6% of the U.S. population to effectively support cancer surveillance efforts [6]. As illustrated by these data [Figure 1], it is evident that the incidence rate of melanoma has increased significantly over time, while the mortality rate has remained relatively stable. Notably, if this trend continues, it is projected that by 2040, melanoma cases will increase by 50% compared to 2020 [5].

**Figure 2:**
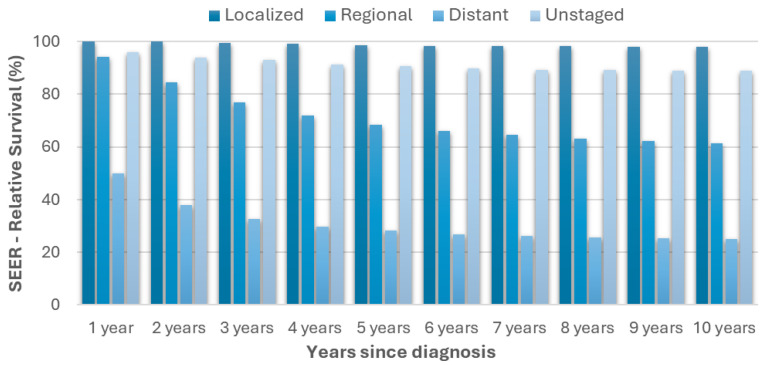
Relative melanoma survival rates over time from diagnosis, differentiated by stage at diagnosis. Reported data were derived from the Surveillance, Epidemiology, and End Results (SEER) program and refer to the period between 2000 and 2019.

Although the incidence of melanoma is still growing substantially, at the present time, a timely diagnosis can grant a 10-year survival rate very close to 100% [Figure 2]. Conversely, advanced melanomas are frequently associated with the presence of metastasis and the long-term prognosis becomes unfavorable. Under this condition, the mortality rate rises to about 75% with variations that are related to multiple epidemiologic factors, such as age, sex, and geography [7,8].

To rationally identify the best therapeutic plan, melanomas are classified at diagnosis based on tumor thickness, the presence or absence of ulceration, extension of metastasis at regional lymph nodes, nearby non-nodal regions, or distant sites [9]. When applicable, the first choice, and most efficient therapeutic approach for the treatment of melanoma, is a surgical excision [10]. To expand the therapeutic options, in 1975 the Food and Drug Administration approved the first chemotherapeutic agent for the systemic treatment of melanoma, the alkylating agent dacarbazine, either alone or in combination with interferon alpha. However, these protocols are not effective in increasing the life expectancy of patients significantly; currently, the use of dacarbazine (or the related telozolomide) is preserved only for unresectable melanomas at stage III and stage IV [11].

At present, the therapeutic strategies are tailored to the cancer stage at diagnosis and the presence of genetic mutations, particularly those occurring at *BRAF*, and they are further adjusted based on the patient’s response. Noteworthy, these mutations, as well as their inhibition, alter the metabolic profile of the cell. This can pave the way for the identification of metabolic biomarkers, to be used to predict the pharmacological response to the treatment in preclinical models [12]. Only in the last decade, twelve new drugs for unresectable melanoma have been approved [13]. In addition, combined therapies are used along neoadjuvant protocols, where one or more of the following treatments are used before surgery to shrink the tumor, and adjuvant therapies, where the drugs are used after surgery to reduce the risk of recurrence.

Standard approved treatments for melanoma include:

- Radiotherapy with high-energy waves is proposed as adjuvant or palliative therapy for the management of melanoma, albeit it is relatively radioresistant [14].
- Immunotherapy that comprises treatment for the inhibition of Interleukin-2 (IL-2), Programmed cell death protein 1 (PD-1) and Programmed death-ligand 1 (PD-L1), Cytotoxic T-lymphocyte-associated protein 4 (CTLA-4) and Lymphocyte Activation Gene 3 (LAG-3) [15].
- Targeted therapies that are designed against mutated BRAF, MEK and c-kit, [10,16].

As anticipated, these therapeutic approaches are usually employed in combination, as multimodality treatments, to overcome resistance mechanisms and to enhance the effectiveness of the treatment. Indeed, even with the most recent options, a single chemotherapeutic agent presents a low response rate ranging from between 10–20% [11], while combined therapies demonstrate significantly higher efficacy. As an example, the current standard treatment for therapy of melanoma expressing mutated *BRAF* [17] is a combination of a CTLA-4 inhibitor (ipilimumab) and a PD-1 inhibitor (nivolumab) that grants generally high responsiveness (≈57%). Unfortunately, it is characterized also by an important toxicity profile since it causes from severe to life-threatening consequences in 55% of the patients [18]. Other drug combinations are currently recommended to treat melanoma at different stages, and even more are under investigation with some of them currently in phase II or III [14].

Despite these promising developments, the summarized data clearly indicate that the current pharmacological options do not fulfill all needs. Addressing these gaps requires the development of innovative therapeutic options. Their design and optimization involve several challenges, foremost the understanding of the molecular mechanisms behind the development and sustainment of melanoma.

## 2. Non-Coding RNA

The original Watson and Crick central dogma of biology linked RNA functions solely to protein synthesis, either as a template (mRNA) or as a functional component to support translation (rRNA and tRNA). Consistently, for two decades, RNA was considered merely an unstable intermediate along protein production, and any DNA that did not code for RNA was dismissed as “junk” DNA. This perspective changed in the 1980s when experiments in E. coli demonstrated that the transcript of the *MICF* gene was directly involved in the regulation of the gene expression [19]. This finding, for the first time, highlighted RNA as a biomolecule capable of interacting with both nucleic acids and proteins to perform a wide range of regulatory functions beyond coding. The regulatory roles of non-coding RNAs (ncRNAs) were further confirmed by the Human Genome Project (HGP). By 2003, when the human genome was fully sequenced, it became clear that only a small fraction of the genome encodes mRNA, while the vast majority is transcribed into RNA with no protein-coding functions yet plays critical roles in regulating cellular processes [20,21].

**Figure 3:**
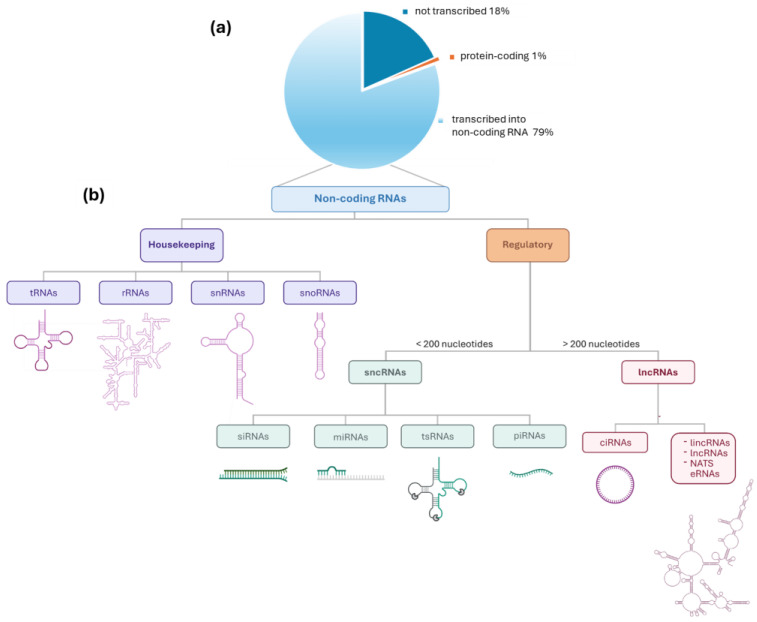
(**a**) Fractions of human genome that are not-transcribed, that codify for proteins- or for non-coding RNAs as derived from ENCODE project data; (**b**) distribution of non-coding RNAs between housekeeping RNA and regulatory RNA and their further classification into sub-classes.

At present, thanks to recent technical advancements, the classification and annotation of genes have been significantly improved and extended. This knowledge is now integrated into the ENCODE project [22], a public resource where all the so-far-identified functional elements of the human genome are classified. This project confirmed that the output originally highlighted by the HGP: ≈80% of the genome is biochemically active, but only a small fraction (≈1%) corresponds to coding exons [Figure 3a] [22,23]. The products of the large population of non-coding genes are ncRNAs transcripts which represent a family of elements with variegated cellular functions and mechanisms of action.

As summarized in Figure 3, at the first level we can cluster non-coding genes into two main categories. The first one corresponds to the family of housekeeping RNAs, i.e., transfer RNAs (tRNA), ribosomal RNAs (rRNAs), small nuclear RNAs (snRNAs), and small nucleolar RNAs (snoRNAs). The second one is the family of the regulatory RNAs, which include short non-coding RNAs (sncRNAs, less than 200 residues) and long non-coding RNAs (lncRNAs, longer than 200 residues). These two groups are further divided into subclasses based on their different main mechanisms of action. Briefly, sncRNAs include micro RNAs (miRNAs), tRNA-derived small RNAs (tsRNAs), small interfering RNAs (siRNAs), and PIWI-interacting RNAs (piRNAs), while the lncRNAs group comprises long intergenic RNA (lincRNA), natural antisense transcripts (NATs), enhancer RNAs (eRNAs), and circular RNAs (circRNAs) [24]. The molecular mechanisms by which non-coding RNAs (ncRNAs) regulate cell fate are diverse and often involve complex interactions between different types of ncRNAs, thus amplifying the strategies they exploit to modulate cellular pathways.

## 3. Correlation of lncRNA and Cancer

As mentioned above, lncRNAs are defined as transcripts longer than 200 nucleotides that do not code for proteins. Like mRNA, they are predominantly transcribed by RNA polymerase II (Pol II) [25].

**Figure 4:**
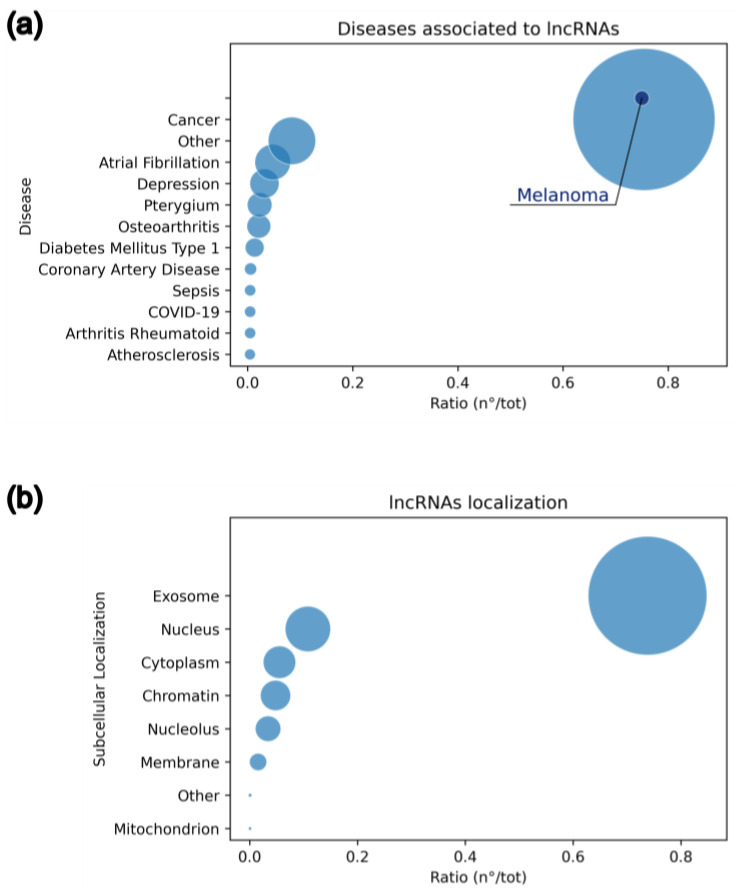
Bubble chart of (**a**) functional relevance of lncRNAs among diseases and (**b**) lncRNAs distribution among cellular compartments as derived from LncRNADisease and RNALocate, respectively.

These biomolecules play a crucial role in the development of several diseases, including cancer, cardiovascular conditions (such as cardiac hypertrophy, ischemic stroke, hypertension, and atherosclerosis), lung diseases (e.g., asthma), metabolic disorders (such as intestinal inflammation, gut microbiota homeostasis, diabetes, and obesity), neurodegenerative diseases (including Alzheimer’s and Parkinson’s), and viral infections (e.g., SARS-CoV-2, influenza A, and hepatitis C) [26,27]. Given the societal impact of these diseases, the experimentally validated associations between lncRNAs and various conditions are currently collected in the online publicly available database LncRNADisease, the v3.0 version of which was released in 2024 [28]. From this database, we retrieved 255 distinct pathologies linked to lncRNAs. As shown in Figure 4a, the most significant associations are with cancer-related diseases, with melanoma representing 1% of the cases.

At a first level, the role of lncRNAs in these diseases can be associated with their differential expression levels. Indeed, some lncRNAs are up or downregulated in cancerous cells, including melanoma, when compared to healthy tissue [29,30,31,32,33]. Accordingly, lncRNAs hold promise also as diagnostic and prognostic markers in cancer [34].

An additional distinguishing feature of lncRNAs in healthy versus cancer cells is their cellular distribution. Indeed, the ability of lncRNAs to accumulate in different cellular compartments selectively tunes their functions. From the analyses of publicly accessible repositories such as RNALocate [35], a noticeable trend emerged. As reported in Figure 4b, lncRNAs are predominantly localized within exosomes, a property that nicely connects them with the regulation of cell–cell crosstalk. The second site in terms of lncRNA enrichment is the nucleus, followed by the cytoplasm. This differential enrichment is interesting since it suggests that, around these compartments, they can exploit variegated functions according to several mechanisms.

### 3.1. Biomolecules Involved in Functional Interactions with lncRNAs

**Figure 5:**
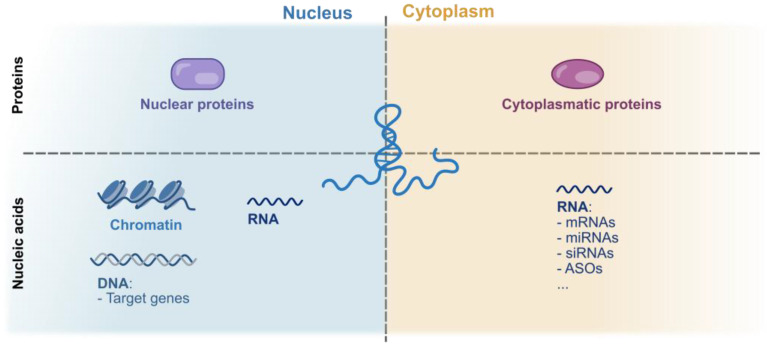
Functional complexes of lncRNAs with biomolecules and their distribution among different cellular compartments.

As introduced above, lncRNAs can contribute to the cancer progression through all the different stages of tumor development that cover proliferation, invasion, and metastasis. This is possible thanks to their ability to interact with RNA, DNA, or proteins according to networks that are differently distributed across various intracellular compartments of proteins [Figure 4 and Figure 5].

**Figure 6:**
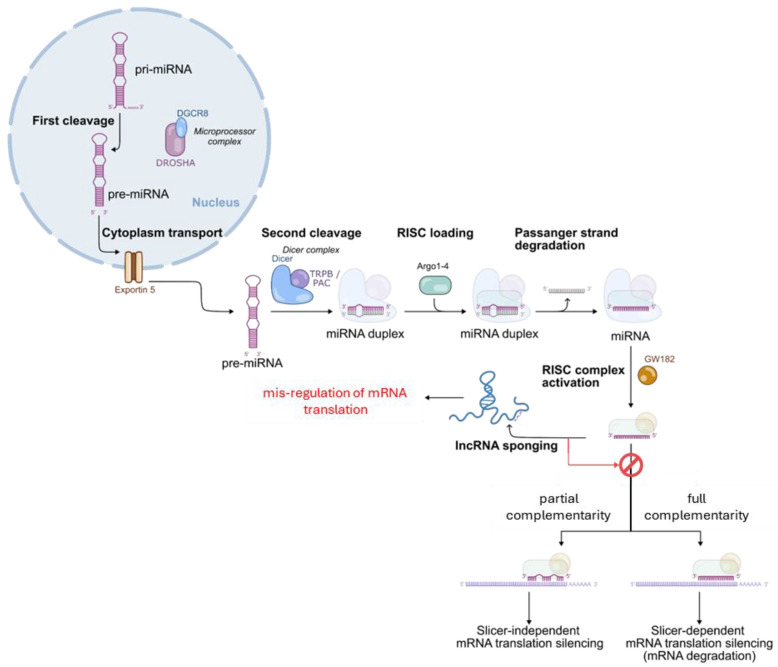
miRNAs biogenesis and point of intervention of lncRNA working according to a sponging mechanism.

In the cytoplasm, lncRNAs can interact with mRNAs, ncRNA, ribosomes, and various cytoplasmic proteins. Among them, a critically relevant functional interaction is the one occurring between lncRNAs and miRNAs [Figure 6] [36]. miRNAs are short non-coding sequences, typically 17–25 nucleotides long. They are primarily responsible for mRNA degradation through either slicer-dependent or independent silencing mechanisms. Although the production of miRNAs begins in the nucleus, it is completed in the cytosol, where they are ultimately released as functionally active single-stranded RNAs. In this compartment, lncRNAs can bind miRNAs through complementary base pairing, a mechanism known as ‘sponging’. As a result, lncRNAs sequester miRNAs from their mRNA targets, thus preserving the corresponding protein production.

**Figure 7:**
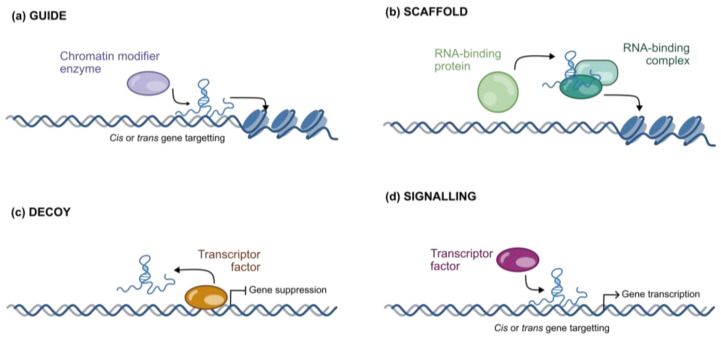
Molecular mechanisms of lncRNAs as epigenetic modulators: (**a**) guide molecules, (**b**) scaffold molecules, (**c**) decoy molecules, and (**d**) signaling molecules.

In the nucleus, lncRNAs can interact with DNA and nuclear proteins too. As a matter of fact, they are directly involved in chromatin remodeling according to variegate molecular mechanisms [37]. As schematically illustrated in Figure 7a, lncRNAs can function as a guide to facilitate the binding of proteins at selected genomic sites. It has been shown that lncRNAs follow this mechanism to regulate the loading of chromatin-modifying enzymes, ultimately enabling the activation or repression of genes either in cis (near to the lncRNA loading site) or in trans (at distant sites or on different chromosomes) [38].

In addition, lncRNAs often act as scaffold molecules [Figure 7b] serving as “anchoring points” that orchestrate and facilitate the assembly of macromolecular complexes. According to this mechanism, lncRNAs mediate the recruitment of RNA–protein complexes (RNPs), which participate in chromatin modification [39].

Furthermore, lncRNAs can function as decoys [Figure 7c] by binding transcription factors and preventing them from activating the expression of target genes. Conversely, lncRNAs can activate gene expression in cis or in trans by recruiting transcription factors, thereby acting as signaling molecules [Figure 7d] [37].

It should be emphasized that, in melanoma, the epigenetic functions of lncRNAs according to all the above-described pathways have not been fully confirmed.

### 3.2. Issues About lncRNA Detection and Functional Validation

Today, providing a comprehensive description of lncRNA functions and mechanisms of action is proving more challenging than initially anticipated. This complexity stems not only from the diversity of their functions and the variability of their mechanisms, but also from additional factors.

One of them derives from the possible expression of multiple isoforms for each lncRNA due to a different transcription starting site (TSS), different splicing, cleavage, polyadenylation, and post-transcription modifications [40,41]. As a result, GENCODE (v26) [42] annotated 20,310 long non-coding RNA genes that led to the production of 59,927 different lncRNAs.

Another issue is related to lncRNA abundance. In reference to mRNA, lncRNAs undergo less efficient splicing events, but their expression levels are generally lower and exhibit greater tissue- and cell-specific variability [43,44]. Furthermore, their expression can be differently regulated along cell stages (i.e., differentiation vs. development) and influenced by environmental factors like stress, drugs assumption, hypoxia, and other physiopathological conditions [45,46,47,48,49,50,51,52]. Due to this heterogeneity, several lncRNAs are often underrepresented in bulk tissue and thus not detected by the most frequently applied sequencing techniques. Therefore, most of the studies reported so far lack proper correlation of molecular mechanisms to a single lncRNA isoform. This creates evident bias in the lncRNA-function associations reported in the currently available databanks and negatively impacts our understanding of their regulatory mechanisms [35]. As proof, in melanoma, only for two lncRNAs, ANRIL (CDKN2B-AS1—Antisense Non-coding RNA in the INK4 Locus) and CRNDE (Colorectal Neoplasia Differentially Expressed), the associated isoforms have been identified and characterized [53,54].

Fortunately, this picture is rapidly evolving. The use of advanced techniques, such as RNA-seq [55], single-molecule real-time sequencing [56] or nanopore sequencing [57], are improving the detection and characterization of lncRNAs as well as the identification of their isoforms. Soon, the lowering of detection limits for lncRNAs is expected to provide a clearer understanding of their differential distribution across cellular compartments. This progress will ultimately enable the design of accurate descriptors to link expression levels, localization, and binding partners to their functional outcomes.

## 4. Validated lncRNA Functional Pathways Relevant in Melanoma

The intricate complexity of lncRNA features mirrors the multiple interconnected mechanisms they play in inducing and supporting melanoma. As a result, identifying a lncRNA as a promising target for innovative genomic approaches is a challenging task. With the aim of providing researchers with a tool that might assist them along this selection process, we used available datasets to cluster those lncRNAs known to support melanoma development according to validated parameters (Table S1). As criteria, we selected the following: the number of transcripts, the expression level variations in melanoma, and the primary mechanism of action, since they are intrinsically interconnected features. The final output is presented in Table 1. By referring to validated examples, we will discuss how this interplay is crucial and, hopefully, druggable.

### 4.1. Chromatin Remodeling

Chromatin remodeling is closely linked to the regulation of gene expression, with histones playing a central role. Indeed, they can undergo various modifications, such as phosphorylation, ubiquitination, methylation, and acetylation, which determine the repression or activation of the expression at target genes. lncRNAs can influence this process by interacting with chromatin-modifying proteins. Among them, the Protein Regulators of Cytokinesis 1 and 2 (PRC1/2) systems are a complex protein assembly that induces histone H3 methylation to promote heterochromatin formation.

In melanoma, the lncRNA known as MIR31 host gene long non-coding (MIR31HG) is upregulated and it directly binds both genomic regions (i.e., *INK4A* and *MIR31HG*) and Polycomb group (PcG) proteins [59]. In detail, it interacts directly with two subunits of PRC2 (SUZ12 and EZH2) and acts as a guide to promote the binding of PRC2 to chromatin. The observed effect related to MIR31HG expression is the epigenetic silencing of the *INK4B-ARF-INK4A* locus, the reduction of p16^INK4A^, and the progression of melanoma development. The investigation on this pathway allowed to validate p16^INK4A^ as an important tumor suppressor thanks to its ability to induce cellular senescence.

**Figure 8:**
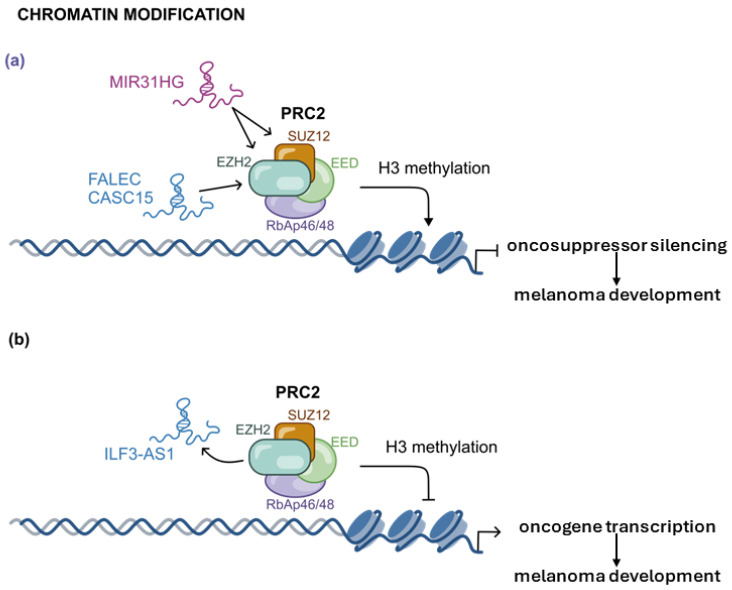
The interaction of selected lncRNAs binding with PRC2 can induce differential chromatin remodeling outputs: (**a**) silencing of oncosuppressors mediated by MIR31HG, FALEC and CASC15 thought the activation of H3 methylation by PRC2, (**b**) suppression of PRC2-mediated H3 methylation by binding ILF3-AS1 at EZH2.

A comparable output is also promoted by FALEC (Focally Amplified lncRNA on Chromosome 1). When upregulated in melanoma cells, it acts as a guide and recruits EZH2 to the p21 promoter. As a result, it epigenetically represses p21 expression, thus contributing to melanoma progression [Figure 8a] [60]. Similarly, the lncRNA CASC15 (Cancer Susceptibility 15) suppresses the expression of the anti-oncogene *PDCD4* (Programmed Cell Death Protein 4) through the recruitment of EZH2 at its promoter. This inhibition promotes cell invasiveness and melanoma progression [Figure 8a] [61].

However, lncRNAs can also contribute to melanoma progression by promoting the transcription oncogene.

Interleukin enhancer-binding factor 3 (ILF3) and ILF3 Antisense RNA 1 lncRNA (ILF3-AS1) are both upregulated in melanoma tissue. The complex formation between ILF3 and ILF3-AS1 enhances the stability and abundance of the lncRNA which, in turn, binds to EZH2. In this case, this cascade prevents the recruitment of PRC2 at the *ILF3* promoter, thereby supporting active transcription of the *ILF3* oncogene. This correlates with the observed increment of proliferation, migration, and invasion by these melanoma cells [Figure 8b] [62].

Besides the ability of lncRNAs to modulate gene expression through the interaction with macromolecular chromatin modifiers, they can influence transcription by directly binding DNA or transcription factors.

**Figure 9:**
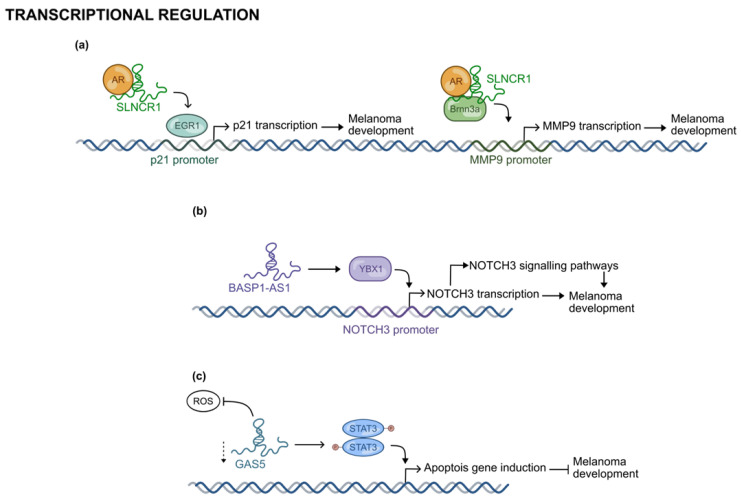
Validated mechanisms of transcriptional regulation exploited by selected lncRNAs in melanoma development (**a**) SLNCR1 activates the expression of different proteins by driving AR to its binding site alone or in combination with other cofactors, (**b**) BASP1-AS1 recruits transcription factors to NOTCH3 promoter (**c**) knockdown of GAS5 prevents the expression of genes required for apoptosis activation.

An intriguing example is represented by SLNCR (Steroid receptor RNA activator (SRA)-like non-coding RNA). As suggested by its name, it interacts directly with the androgen receptor (AR) through a highly conserved sequence that is similar to the one present in the lncRNA SRA1 (Steroid Receptor RNA Activator 1). Thanks to this interaction, SLNCR1 functions as a guide molecule by tethering the androgen receptor (AR) to the EGR1-consensus-motif in the promoter of *CDKN1A*, thereby promoting p21 expression and contributing to oncogenesis [Figure 9a] [63]. Noteworthy, SLNCR possesses also a Brn3a (Brain-Specific Homeobox/POU Domain Protein 3A) domain located near the AR domain. The cooperative formation of the nuclear SLNCR1/AR/Brn3a complex is responsible for the transcriptional activation of matrix metalloproteinase 9 (MMP9). The final increment of MMP9 expression and activity enhances the invasion capability of melanoma cells [Figure 9a] [64]. Recently, SLNCR1 was also associated with SOX5 upregulation promoting Epithelia-to-Mesenchymal Transition (EMT) in human melanoma [65].

Also, the lncRNA BASP1-AS1 (Brain Abundant, Membrane Attached Signal Protein 1 Antisense RNA 1) is more expressed in melanoma tissues and melanoma cell lines compared to healthy skin tissues. In these cells, it epigenetically activates the expression of Neurogenic locus notch homolog protein 3 (NOTCH3) by recruiting the transcript factor YBX1 (Y Box binding protein 1) at the promoter of NOTCH3. The resulting activation of NOTCH-signaling results in the transcription of multiple oncogenes (c-MYC, PCNA, and CDK4) that promote melanoma progression [Figure 9b] [66].

Conversely, GAS5 (Growth Arrest-Specific 5) is a lncRNA downregulated in melanoma tissue compared to adjacent noncancerous tissues. In normal cells, it is implied in the regulation of cell proliferation, of the homeostasis of redox balance and in the induction of apoptosis through the inhibition of Cyclin D1, CDK4, p27, and Blc-2 expression. Consistently, its knockdown induces G1/S progression via STAT3-mediated signaling pathways, thus contributing to melanoma progression [Figure 9c] [67].

### 4.2. Post-Transcriptional Regulation

As an additional level of intervention, lncRNAs can be actively involved in the post-transcriptional processing of mRNA where they can tune mRNA alternative splicing, stability, and localization. These functions can be the result of a “simple” mRNA-lncRNA interaction or can be controlled through more articulated biological processes.

**Figure 10:**
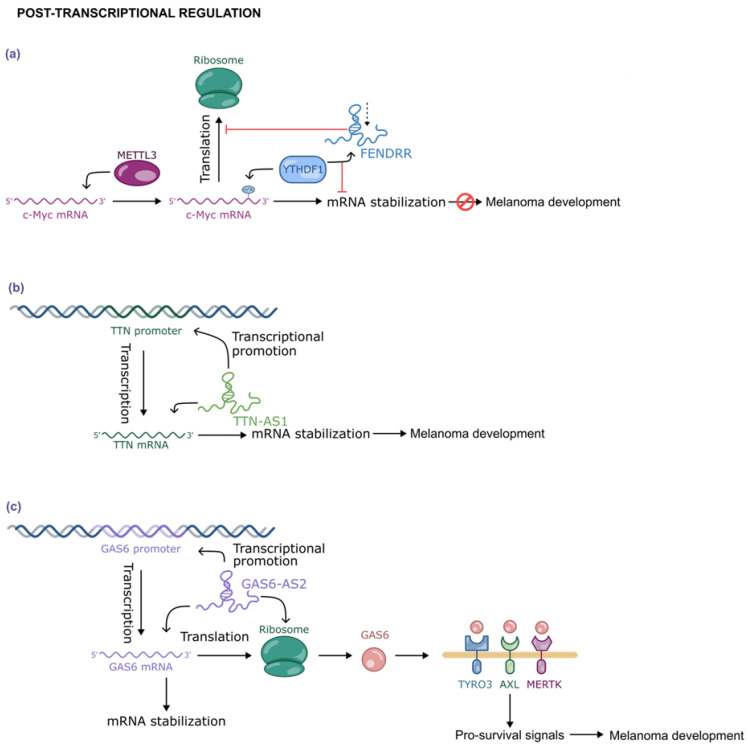
Validated mechanisms of post-transcriptional regulation exploited by selected lncRNAs in melanoma development: (**a**) the downregulation of FENDRR allows YTHDF1 to increase the half-lives of the cMYC mRNA, (**b**) TTN-AS1 and (**c**) GAS6-AS2 increments the synthesis and stability of TTN and GAS6 mRNA, respectively.

An example relevant in melanoma is represented by FENDRR (FOXF1 adjacent non-coding developmental regulatory RNA), a lncRNA that inhibits cell proliferation through a negative post-transcriptional regulation of c-Myc [68,69]. Specifically, the Methyltransferase Like 3 (METTL3) is known to catalyze the m6A modification at the 3′-UTR of the c-Myc mRNA. This modification recruits the m6A readers YTHDF1 (YTH Domain Family Member 1) leading to increased stability of the c-Myc mRNA, thereby promoting tumorigenesis [Figure 10a]. FENDRR can elicit this effect by binding YTHDF. However, in melanoma cells, FENDRR is downregulated, which results in the stabilization of c-Myc mRNA and, consequently, in the promotion of cell cycle progression and melanoma proliferation [Figure 10a].

TTN-AS1 (Titin Antisense RNA 1) is a lncRNA transcribed in the antisense strand of the human titin gene (*TTN*). The upregulation of the lncRNA TTN-AS1 was found to promote the expression of *TTN* acting both at a transcriptional and post-transcriptional level. The result is an accumulation of TTN protein in the cytoplasm that has been proven to be connected to melanoma tumorigenesis and metastasis as well as to immune response [Figure 10b] [70,71].

Another lncRNA upregulated in melanoma cell lines and connected to the tumor progression is GAS6-AS2. It promotes *GAS6* transcription and stabilizes GAS6 mRNA, thus increasing protein production. The resulting cytokine GAS6 binds and activates some tyrosine kinase receptors like TYRO3, AXL, and MERTK (TAM) which are involved in pro-survival signals [Figure 10c] [72].

### 4.3. Translational Regulation

Also moving at the translational level, the functional mechanisms of lncRNA can be variegated covering either interaction with proteins (principally translation initiation factors or RNA-binding protein) and sponging of miRNAs [Figure 6]. The last one is one of the most extensively studied for the potential design of related ASO with therapeutic applications. Few examples are reported to highlight some variations in molecular mechanisms.

MEG3 (Maternally Expressed Gene 3) is a lncRNA that binds miR-208 to promote the transcription of SOX4. In melanoma cells MEG3 is downregulated, miR-208 is overexpressed, and this results in the inhibition of SOX4 transcription. It is proposed that by driving the overexpression of MEG3, it should be possible to suppress melanoma progression and metastasis [73].

Conversely, SNHG5 (Small Nucleolar RNA Host Gene 5) is upregulated in melanoma cells. This lncRNA works as a “sponge” for miR-26a-5p to promote the expression of TRPC3 (Transient Receptor Potential Cation Channel Subfamily C Member 3) ultimately driving proliferation and invasion by the cancer cells [74].

MSC-AS1 (Mesenchymal Stem Cell-Associated Antisense RNA 1) is another lncRNA upregulated in melanoma. It can pair to miR-330-3p but can also bind directly to YAP1. This appears to alter the miR-330-3p/ YAP1 pathway, ultimately supporting proliferation and glutaminolysis of melanoma cells [75].

Worth mentioning is the fact that translational regulation by lncRNA does not always require miRNA sponging. The lncRNA TINCR (Terminal Differentiation-Induced Noncoding RNA) is an example of cytoplasmatic lncRNA downregulated in melanoma. Low levels of TINCR are associated with the efficient expression of ATF4 (Activating Transcription Factor 4), a protein involved in protein synthesis and tumorigenesis, while high levels of the lncRNA drive MITF (melanocyte inducing transcription factor) expression, which preserves the epithelial state of the cells. This occurs because TINCR prevents the binding of target mRNAs to the ribosomes, thereby inducing translational reprogramming [76].

### 4.4. Post-Translational Regulation

lncRNAs are frequently involved in protein modification at post-translational level thereby inducing significant alterations in protein folding, stability, and activity.

**Figure 11:**
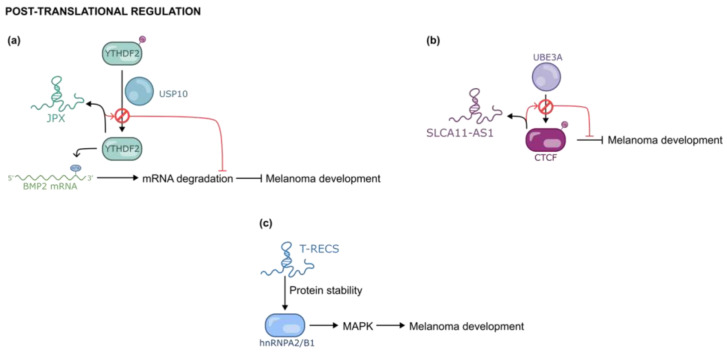
Mechanisms of post-translational regulation exploited by selected lncRNAs in melanoma development: (**a**) JPX prevents the deubiquitylation of YTHDF2; (**b**) SLC11-AS1 prevents the ubiquitination of CTCF and (**c**) T-RECS activates MAPK signaling by increasing the stability of hnRNPA2/B1.

YTHDF2 is a protein involved in the modulation of m6A-modified mRNAs, orchestrating the cytoplasmatic degradation of several mRNA, including the Bone Morphogenetic Protein 2 (BMP2) mRNA. The lncRNA Just Proximal to Xist (JPX) interacts with YTHDF2 and prevents it binding to the ubiquitin-specific protease 10 (USP10). Accordingly, YTHDF2 is not deubiquitylated anymore, and this leads to its degradation. As a result, BMP2 mRNA is preserved and can maintain its beneficial functional control on EMT and cellular stemness [Figure 11a] [77].

SLC7A11-AS1 (Solute Carrier Family 7 Member 11 Antisense RNA 1) is a lncRNA which highly expresses in melanoma. It modulates the levels of CCCTC-binding factor (CTCF), a chromatin-binding factor that participates in transcriptional regulation by orchestrating the interaction between genome domains. Consequently, CTCF controls variegate cellular pathways including those related to DNA repair and cell proliferation. SLCA11.AS1 inhibits the degradation of CTCF by preventing its ubiquitination by Ubiquitin Protein Ligase E3A (UBE3A), thus promoting melanoma progression [Figure 11b] [78].

A recent study identified a novel nuclear-enriched lncRNA named T-RECS that is upregulated in the NRAS/MAPK-dependent melanoma cell line. T-RECS is involved in the enhancing of pro-survival kinases activity and in the increase of hnRNPA2/B1 (Heterogeneous Nuclear Ribonucleoprotein A2/B1) protein stability. The protein hnRNPA2/B1 acts as a pro-oncogenic regulator of MAPK signaling. The selective targeting of this lncRNA with an ASO has been reported to reduce the progression of melanoma cells [Figure 11c) [79].

## 5. Implications of lncRNA Localization on Regulatory Network in Melanoma

As introduced above, lncRNAs can work as epigenetic, post-transcriptional, translational, and post-translational modulators to regulate gene expression through different mechanisms. Moreover, they can trigger distinct cellular pathways not only according to interaction networks they actively establish with proteins, RNA, and DNA, but also as a result of their preferential accumulation at specific cellular loci.

The previously described MIR31HG nicely illustrates this option. When upregulated, it reduces the expression of p16^INK4A^, a protein involved in senescence, by an epigenetic mechanism that cooperatively requires the nuclear protein complex PRC2 [59]. As expected, the silencing of this lncRNA results in cell senescence. However, paradoxically, this lncRNA is also over-expressed during BRAF-induced senescence. This contradictory outcome is related to a subcellular redistribution of MIR31HG. Indeed, in normal conditions, MIR31HG is located both in the nucleus and the cytoplasm, with a slight enrichment in nuclear fraction. Conversely, during BRAF-induced senescence, this lncRNA is translocated into the cytoplasm by an Aly-dependent RNA export pathway. As a result, its absence in the nucleus prevents its interaction with PRC2, thus resulting in senescence inhibition. This example validates the intracellular distribution as a tool to balance different functions of MIR31HG.

The following examples will summarize how lncRNAs preferentially work within different compartments.

### 5.1. Exosomes

Exosomes are defined as a subset of extracellular vesicles (EVs) with an average diameter of ~100 nm that are produced by most eukaryotic cells to be released in the extracellular environment [80]. Exosomes are extensively involved in cell-to-cell communication, although their roles are further extended to the remodeling of a tumor microenvironment and the maintenance of cellular homeostasis [81]. In line, exosomes are implied in melanoma pathogenesis, growth, and metastasis formation [82,83].

As far as neogenesis is concerned, it has been shown that melanoma initiation can be induced by exosomes released from bone marrow mesenchymal stem cells (BMSCs). These extracellular vesicles (EVs) contain the lncRNA nuclear paraspeckle assembly transcript 1 (NEAT1). When they are internalized by macrophage, NEAT1 is released, and it can interact with several miRNAs. Among them, miR-374 is a validated target. This interaction results in an impairment of the miR-374a-5p/LGR4/IQGAP1 axis that leads to the promotion of M2 macrophage polarization, ultimately driving melanoma progression [84].

Exosomes released from melanoma cells can also promote melanoma progression. As an example, this occurs when they deliver the lncRNA Gm26809 into normal fibroblasts. This activates their reprogramming into cancer-associated fibroblasts (CAFs) with an upregulation of CAF markers such as α-smooth muscle actin (α-SMA) and fibroblast activation protein (FAP) [85]. This pathway facilitates melanoma cell proliferation and migration.

In addition, Gm33149, a lncRNA upregulated in highly metastatic melanoma stem cells, can be transferred via exosomes into low-metastatic non-stem cells (OL). After Gm33149 internalization, these cells acquire a more aggressive metastatic phenotype as a result of its pairing (sponge) to miR-5623-3p. Such interaction silences the miRNA and causes the activation of the Wnt signaling pathway that is responsible for the enhancement of cell migration, invasion, and metastatic behavior [86].

### 5.2. Nucleus

Looking at the frequency distribution [Figure 4b], the preferential localization of intracellular lncRNAs is the nucleus. In this subcellular compartment, as we have already widely discussed, they can interact directly with nucleic acids and proteins, such as chromatin-modifying complexes and transcription factors, leading to chromatin remodeling, and transcriptional and post-transcriptional regulation [48].

Additional components of the interchromatin space that deserve consideration are the subnuclear bodies known as paraspeckles. These structures form through RNA–protein interactions, and their role in regulating gene expression is currently the subject of extensive research [87]. A lncRNA actively involved in nuclear paraspeckles formation is NEAT1. Its presence is strongly associated with tumorigenesis according to its ability to enhance cell survival. Unexpectedly, p53, which is known as “the guardian of the genome”, is functionally related to NEAT1. Specifically, p53 stimulates the formation of NEAT1-loaded paraspeckles; these help to maintain genomic integrity by preventing DNA damage during replication stress. Therefore, paraspeckles are crucial for cell survival under critical physiological conditions, such as replication stress [88].

### 5.3. Cytoplasm

Cytoplasmatic lncRNAs are less abundant than their nuclear counterparts [Figure 4b], but their relevance in melanoma is not reduced thanks to their function as regulatory elements during transcription, post-transcription, translation, and post-translation [25,89,90,91]. For drug design, this is an ideal condition. Indeed, the cytosolic localization of an oncogenic lncRNA is a remarkable advantage since it is more accessible for therapeutic intervention.

An example of lncRNA primarily located in the cytoplasm and upregulated in melanoma cell lines is LENOX (LINC00518—lincRNA-enhancer of oxidative phosphorylation). It promotes the association of Ras-related protein 2 (RAP2C), a GTPase, with DRP1 (Dynamin-Related Protein 1), a mitochondrial fission regulator. This interaction supports mitochondrial fusion and oxidative phosphorylation. In addition, LENOX is responsible for the resistance to Mitogen-Activated Protein Kinase (MAPK)-inhibition. Overall, the targeting of LENOX with antisense oligonucleotides (ASOs) combined with the silencing of RAP2C and MAPK-inhibitors is expected to also eradicate MAPK-resistant melanoma cells [92].

Nevertheless, several lncRNAs can localize in both the nucleus and cytoplasm, although some of them are functionally active in only one compartment. An example is THOR (Testis-associated Highly-conserved Oncogenic long non-coding RNA) which is known to bind the Insulin-like Growth Factor 2 mRNA-Binding Protein 1 (IGF2BP1). IGF2BP1 is exclusively present in the cytoplasmatic peri-nuclear region where it stabilizes a set of mRNAs as follows: IGF2, CD44, KRAS, ACTB, PABPC1, GLI1, MYC, MAPT, CTNNB1, PPP1R9B, BTRC, PTEN, and H19. THOR contributes to the stabilization of IGF2BP1, although with a still unknown mechanism [93].

### 5.4. Mitochondria

Only a minimal percentage of lncRNAs are localized in mitochondria. Among them, an example is SAMMSON (Survival Associated Mitochondrial Melanoma Specific Oncogenic Non-coding RNA), which is one of the most relevant lncRNAs in melanoma, where it plays pivotal roles in the maintenance of oxidative phosphorylation, opposing glycolysis, thereby sustaining the growth of cancer cells [94]. The largest amount of SAMMSON is localized in the cytoplasm with only a modest fraction in the mitochondria. Nevertheless, within this compartment, SAMMSON can interact with p32, a protein upregulated in tumors. The silencing of SAMMSON results in the reduction of p32 within mitochondria. This condition perturbs mitochondrial functions and leads to mitochondria-dependent apoptosis in melanoma cells [95].

## 6. Conclusions

At present, the relevance of lncRNAs in the initiation and development of various pathologies, including melanoma, is well recognized. Since they can actively participate in complex regulatory networks, the molecular mechanisms that relate most lncRNAs to these functions are still under investigation. Nevertheless, they offer exciting prospects for advancing our understanding of the induction and the development of melanoma. We expect they will shortly provide new perspectives to improve the management of diseases, as well as the setting up of new therapeutic approaches.

This potential is rapidly expanding thanks to the development of new methods for their identification (i.e., CHART, RAP, RIP, ChIRP, and CLIP) and new technologies for their sequencing (i.e., LIGR-seq, Gro-seq, and BRIC-seq). In addition, advances in bioinformatics and new machine learning approaches are helping to predict lncRNA interactions with proteins or other biomolecules. Last but not least, online databases such as LncRNADisease v3.0 [28], Lnc2Cancer 3.0 [58], and GENCODE [42] provide free access to a wealth of information spanning experimental validations and functional annotations to clinical associations. All these data are critically relevant from a medicinal chemistry point of view. Indeed, only a detailed characterization of the relevant pathways and the molecular mechanisms behind them can support an efficient drug discovery program.

However, properly scanning such a vast amount of information can be challenging. Moreover, the examples discussed in this review highlight how lncRNAs can influence cell behavior at any stage of melanoma through the regulations of chromatin architecture, transcription, post-transcription, signal transduction, and post-transduction events. This plastic profile represents an additional level of complexity in the understanding of lncRNAs and provides great opportunities. Indeed, lncRNA expression profiles can be exploited for the design of proper biomarkers able to identify melanoma at a very early stage and to suggest the best therapeutic approach [12,34]. This will drive towards a personalized approach, which is expected not only to further increase the survival rate but also to reduce unneeded treatments with an overall benefit for the patient and with reduced costs.

This scenario is also relevant for therapeutic purposes. The inhibition of upregulated lncRNAs, or the inductions of the silenced ones, has direct and significant positive effects on controlling cell progression and/or invasiveness in melanoma. Again, their activity profile appears to be influenced both by their tissue and cellular compartment expression. Although extremely complex, this picture offers great opportunities for the identification of new targets for the development of innovative treatments for melanoma. In this field, it is rapidly expanding the use of nucleic acid-based drugs that recognize the selected lncRNA based on primary sequence complementarity. While this approach offers a sort of “simple” design for the selective target recognition, it appears to be highly demanding in terms of optimization when the drug has to be modified to grant proper biostability and accumulation at the right physiological compartment or when the design of proper delivery systems must be considered [96,97]. These steps are expensive, mostly in terms of time. This further reinforces the critical relevance of the target selection step to successfully provide the best therapeutic options for the patient as soon as possible.

## Figures and Tables

**Table 1:** Name, number of transcripts, expression levels, main reported mechanism of action, reported localization, and primary citation of lncRNAs studied in melanoma cell lines. Data were derived from LncRNADisease v3.0, [28] and Lnc2Cancer 3.0 [58]; the number of transcripts from GENCODE [42].

Name	n° of Transcripts	Regulation	Main Mechanism of Action	Reported Localization	Primary Citation (PMID Code)
ANRIL	Not found in .gff3	Differential expression	-	-	28653984
Llme23	Not found in .gff3	Differential expression	Binds recombinant and native PSF protein	Nucleus	23618401
CASC2	27	Downregulated	Sponges miR-181a promoting the expression of *PLXNC1*	-	29514220
DIRC3	31	Downregulated	Modulates chromatin structure and inhibits the binding of SOX10 at DIRC3 locus, thus enhancing the expression of *IGFBP5*	Nucleus	31881017
FUT8-AS1	1	Downregulated	Silences p15	-	34094894
GAS5	Not found in .gff3	Downregulated	Inhibits *EZH2* expression by recruiting E2F4 to its promoter	Nucleus	32308561
HAND2-AS1	65	Downregulated	Downregulates *ROCK1*	-	31423160
HCP5	9	Downregulated	Sponges miR-1286 upregulating *RARRES3*	-	31496735
HOTAIR	13	Downregulated	Sponges miR-152-3p to release-MET mRNA resulting in the activation of PI3k/Akt/mTOR signaling pathway	Nucleus and cytoplasm	29156728
ITCH	3	Downregulated	Downregulates *GLUT1* and suppresses glucose uptake	-	31403357
LINC00459	1	Downregulated	-	Cytoplasm	31844121
LINC01550	8	Downregulated	-	-	33609219
LINC-PINT	69	Downregulated	Recruits EZH2 to the promoter of *CDK1*, *CCNA2*, *AURKA*, and *PCNA*, leading to H3K27 trimethylation and epigenetic silencing of target genes	Nucleus	31921860
LINC-ROR	8	Downregulated	-	-	26314857
MEG3	50	Downregulated	Inhibits Wnt signaling pathway by reducing β-catenin and CyclinD1 and rising GSK-3β levels	-	29781534
MIR155HG	2	Downregulated	Sponges miR-485-3p upregulating *PSIP1* expression	Nucleus	34225636
MIR31HG	27	Downregulated	Interacts with SUZ12 causing repression of p16 transcription	Cytoplasm	25908244
NKILA	2	Downregulated	Inhibits NF-kB	-	28123845
PAUPAR	18	Downregulated	Modulates *HES1* expression by inhibiting H3K27 trimethylation	Nucleus	27214741
SSATX	Not found in .gff3	Downregulated	-	-	31352009
TINCR	Not found in .gff3	Downregulated	Sponges miR-424-5p upregulating *LATS1* expression. This activates Hippo signaling, represses the activity of YAP, and the expression of *AXL*, *CTGF* and *CCN1*	Cytoplasm	34542165
AFAP1-AS1	2	Upregulated	Interacts with BRD7 reducing c-Myc expression	-	36777828
ATB	87	Upregulated	Recruits YBX1 to the promoter of *NOTCH3* thus activating the transcription of c-MYC, PCNA, and CDK4	-	29956757
BANCR	5	Upregulated	Regulates activation of MAPK pathway	-	24967732
BASP1-AS1	8	Upregulated	Interacts with YBX1 regulating *NOTCH* transcription	Nucleus	34533860
BLACAT1	Not found in .gff3	Upregulated	Sponges miR-374-5b releasing UHMK1	Nucleus	34708547
CAR10	Not found in .gff3	Upregulated	Sponges miR-125b-5p to inducing *RAB3D* expression	-	32636644
CASC15	Not found in .gff3	Upregulated	Downregulates *PDCD4* expression through EZH2 recruitment to increase H3K27me3 level	Nucleus	30013768
CCAT1	Not found in .gff3	Upregulated	Sponges miR-33a	-	28409554
CD27-AS1-208	1	Upregulated	Interacts with STAT3	Nucleus	35096622
CRNDE	30	Upregulated	Sponges miR-205	-	30257602
DBH-AS1	1	Upregulated	Sponges miR-223-3p increasing *EGFR* expression activating Akt/GLUT1 pathway	Cytoplasm	32744696
DSCAM-AS1	4	Upregulated	Sponges miR-136	Cytoplasm	31002140
DUXAP8	9	Upregulated	Sponges miR-3182 releasing NUPR1	-	33981357
FAL1	Not found in .gff3	Upregulated	Binds BMI1 to modulate CDKN1A and repressing p21	Nucleus	25203321
FALEC	1	Upregulated	Represses p21 through recruiting EZH2 to its promoter 1	Nucleus	29196104
FGD5-AS1	10	Upregulated	-	-	32997827
FOXC2-AS1	1	Upregulated	Silences p15 by recruiting EZH2	Cytoplasm	32964984
FOXD2-AS1	1	Upregulated	-	Cytoplasm	30426532
FOXD3-AS1	20	Upregulated	Sponges miR-127-3p	Cytoplasm	32354225
GAS6-AS2	Not found in .gff3	Upregulated	Upregulates GAS6 expression and secretion and activates AXL/AKT/ERK signals	-	31162889
GAS6-DT	3	Upregulated	Activates GAS6/AXL/AKT/ERK signals	-	31162889
Gm26809	Not found in .gff3	Upregulated	Enhances CAF markers (α-SMA and FAP)	Cytoplasm	31544590
Gm33149	Not found in .gff3	Upregulated	Sponges with miR-5623-3p activated the Wnt signaling pathway	Cytoplasm	38072970
H19	15	Upregulated	Regulates the EMT-related gene expressions	-	29950863
HCG18	42	Upregulated	Sponges miR-324-5p upregulating CDK16 expression	Nucleus and cytoplasm	35273726
HOXD-AS1	10	Upregulated	Suppresses the expression of *RUNX3* via binding to EZH2	Nucleus	29312805
ILF3-AS1	Not found in .gff3	Upregulated	Inhibits the binding of EZH2 to *ILF3* promoter and activates *ILF3* transcription by inducing euchromatin formation	Nucleus	30588088
JPX	85	Upregulated	Interacts with YTHDF2 and inhibits its deubiquitination leading to BMP2 mRNA stabilization and activation of AKT phosphorylation	-	38441563
KCNQ1OT1	85	Upregulated	Sponges miR-153	-	29667930
LENOX	Not found in .gff3	Upregulated	Promotes association of the RAP2C GTPase with mitochondrial fission regulator DRP1, increasing DRP1 S637 phosphorylation, mitochondrial fusion, and oxidative phosphorylation	Cytoplasm	36214632
LHFPL3-AS1	7	Upregulated	Sponges miR-580-3p to rise *STAT3* expression, resulting in the activation of the JAK2/STAT3 signaling pathway	Cytoplasm	32753471
LICN00518	Not found in .gff3	Upregulated	Sponges miR-204-5p upregulating *AP1S2* expression	Cytoplasm	31712557
LICN00520	Not found in .gff3	Upregulated	Sponges miR-125b-5p releasing EIF5A2	Nucleus and cytoplasm	32466797
LINC00173	5	Upregulated	-	-	35239877
LINC00511	107	Upregulated	Sponges miR-625-5p upregulating *PKM2* expression	Nucleus and cytoplasm	34218270
LINC00665	35	Upregulated	Sponges miR-224-5p to upregulate *VMA21*	-	33247967
LINC00673	Not found in .gff3	Upregulated	-	Nucleus	27210747
LINC00963	65	Upregulated	Sponges miR-608 promoting the expression of *NACC1*	-	30180950
LINC01234	17	Upregulated	-	Nucleus and cytoplasm	34218270
LNCOC1	9	Upregulated	Sponges miR-124	-	35502349
lncRNA-ATB	Not found in .gff3	Upregulated	Sponges miR-590-5p to release YAP1 mRNA	-	29956757
LNMAT1	Not found in .gff3	Upregulated	Inhibits *CADM1* expression by recruiting EZH2 at its promoter r	-	31334110
LUADT1	1	Upregulated	Sponges miR-28-5p to upregulate *RAP1B*	-	32191497
MALAT1	17	Upregulated	Sponges miR-34a	-	31101802
MHENCR	4	Upregulated	By sponging miR-425 and miR-489, increases *IGF1* and *SPIN1* expression and activates PI3K-Akt pathway	Cytoplasm	28123636
MIR205HG	17	Upregulated	Sponges miR-299-3p and upregulates *VEGFA* expression	-	33535182
MIR4435-2HG	108	Upregulated	Sponges miR-802 that directly targets *FLOT2*	-	32196611
MIRAT	Not found in .gff3	Upregulated	Binds to the MEK scaffold protein IQGAP1 modulating MAPK pathway	Cytoplasm	30026510
MSC-AS1	14	Upregulated	Sponges miR-302a-3p, recruits IGF2BP2 and increases *LEF1* expression	Cytoplasm	34218464
NCK1-DT	9	Upregulated	Sponges miR-526b-5p upregulating *ADAM1* expression	Cytoplasm	34247598
NEAT1	9	Upregulated	Interacts with miR-374a-5p/LGR4/IQGAP1 axis	Exosomes	32096166
NORAD	1	Upregulated	Sponges miR-205 upregulating *EGLN2* expression	-	30843652
NR2F1-AS1	75	Upregulated	Sponges miR-493-5p and upregulates *GOLM1* expression	Cytoplasm	33822440
PANDAR	Not found in .gff3	Upregulated	Regulates EMT	-	31938355
PEG10	Not found in .gff3	Upregulated	Enhances cyclinD1 and CDK4 expression and sponges miR-33a	-	31318088
POU3F3	Not found in .gff3	Upregulated	Downregulates *MEG3*	-	35201451
PVT1	190	Upregulated	Binds to EZH2 and regulates the expression of miR-200c	-	29286144
RMEL3	1	Upregulated	-	-	30457212
RP11-705C15.3	Not found in .gff3	Upregulated	Sponges miR-145-5p activating NRAS/MAPK signaling axis	Cytoplasm	33311650
SAMMSON	28	Upregulated	Disrupts vital mitochondrial functions	Mitochondria	27008969
SLC7A11-AS1	5	Upregulated	Reduces CTCF degradation by inhibiting its ubiquitination by UBE3A	Cytoplasm	38187043
SLCO4A1-AS1	3	Upregulated	Sponges miR-1306-5p upregulating *PCGF2* expression		35427425
SLNCR	Not found in .gff3	Upregulated	Recruits AR to EGR1 bound genomic loci to inhibit p21 transcriptional activation	Nucleus	31116991
SLNCR1	Not found in .gff3	Upregulated	Binds AR, and Brn3a for transcriptional activation of MMP9	Nucleus	27210747
SNHG12	19	Upregulated	Sponges miR-199b upregulating *ETS1*, *PXN*, *JAG1*, and *DDR1* expression	-	35280401
SNHG16	18	Upregulated	Sponges miR-205-5p upregulating *PAK2* expression	Nucleus and cytoplasm	35983126
SNHG17	118	Upregulated	Enhances the PI3K/AKT signaling pathway		31599425
SNHG5	89	Upregulated	Binds miR-26a-5p thus promoting *TRPC3* expression	-	30636880
SNHG8	15	Upregulated	Sponges miR-656-3p upregulating *SERPINE1* expression	Cytoplasm	35156513
SPRY4-IT1	10	Upregulated	Sponges miR-22-3p thus enhancing MAPK pathway	-	31933852
SRA	1	Upregulated	Mediates p38 activation	-	31945347
THOR	10	Upregulated	Enhances IGF2BP1 mRNA stabilization activity	Nucleus and cytoplasm	29245011
T-RECS	Not found in .gff3	Upregulated	Enhances pro-survival kinases activity and increases hnRNPA2/B1 stability	Nucleus	38077055
TTN-AS1	81	Upregulated	Increases the activity of TTN promoter and increases the stability of TTN mRNA	Nucleus	32820147
TUG1	Not found in .gff3	Upregulated	Sponges miR-129-5p liberating AEG1 thus enhancing the expression of Bcl-2, MMP-9, and cyclin D1	-	29543785
UCA1	45	Upregulated	Sponges with miR-28-5p liberating HOXB3	-	30988802
ZEB1-AS1	12	Upregulated	Sponges miR-1224-5p	-	30651872
ZFAS1	14	Upregulated	Sponges miR-150-5p liberating RAB9A	-	30889053

## Supplementary Materials

The following supporting information can be downloaded at: https://www.mdpi.com/article/10.3390/ijms26052126/s1.

## Abbreviations

The following abbreviations are used in this manuscript:

lncRNAs	Long non-coding RNAs
SEER	Surveillance, Epidemiology, and End Results
HGP	Human Genome Project
